# Flexor Carpi Radialis Tendon Rupture Associated With a Distal Radius Fracture: A Case Report Highlighting Preoperative Three-Dimensional CT Findings

**DOI:** 10.7759/cureus.108058

**Published:** 2026-04-30

**Authors:** Shun Yasukochi, Kimitaka Nakamura, Takahiro Hamada, Akihiko Inokuchi, Teiyu Izumi, Ryuta Imamura, Yu Setoyama, Keisuke Hoshiko, Daisuke Hirano, Takeshi Arizono

**Affiliations:** 1 Department of Orthopaedic Surgery, Kyushu Central Hospital of the Mutual Aid Association of Public School Teachers, Fukuoka, JPN

**Keywords:** 3d computed tomography, distal radius fracture, flexor carpi radialis tendon rupture, tendon injury, wrist trauma

## Abstract

Flexor carpi radialis (FCR) tendon rupture associated with a distal radius fracture is a rare entity. We report a case of a 48-year-old man who sustained a Colles-type distal radius fracture complicated by an FCR tendon rupture at the time of injury. The rupture was first identified intraoperatively during open reduction and internal fixation. Radiographs demonstrated a prominent volar radial third fragment along the anatomical course of the FCR tendon. Retrospective three-dimensional (3D) reconstruction of preoperative CT images using soft-tissue settings clearly revealed discontinuity of the tendon. This case suggests that clinicians should maintain a high index of suspicion for FCR injury whenever a volarly protruding fragment is observed. Furthermore, 3D-CT reconstruction focusing on the tendon course provides a practical and accessible tool that may facilitate preoperative recognition of tendon discontinuity and improve surgical preparedness.

## Introduction

Distal radius fractures are among the most common injuries treated by orthopedic surgeons [1]. While complications of the extensor tendons - particularly delayed rupture of the extensor pollicis longus - are well documented [2], acute flexor tendon ruptures at the time of injury are rare [3]. Rupture of the flexor carpi radialis (FCR) tendon associated with a distal radius fracture is exceptionally uncommon [4], and its clinical features and diagnostic indicators have not been well described [5]. In particular, the potential association between specific fracture patterns, such as a volar protruding bone fragment, and FCR tendon injury remains poorly recognized. We report a rare case of FCR tendon rupture associated with a Colles-type distal radius fracture, highlighting the importance of suspecting this injury when characteristic bone fragments are present and the potential role of three-dimensional (3D) CT reconstruction in facilitating its preoperative detection.

## Case presentation

**Figure 1:**
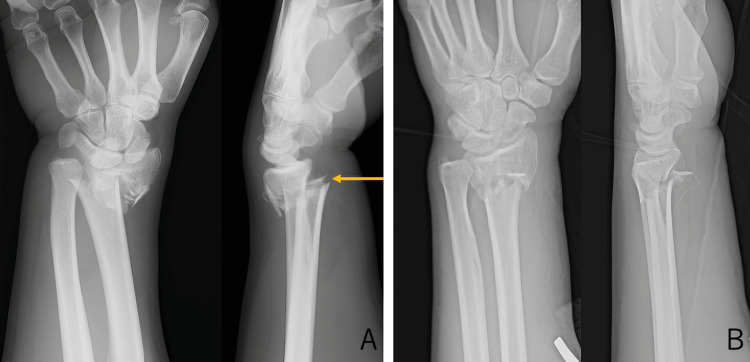
Preoperative radiographs of the left wrist A: Initial plain radiograph at presentation demonstrating a Colles-type distal radius fracture. A prominent radial volar third fragment is visible (arrow). B: Post-reduction radiograph obtained on the day of injury showing improved alignment after closed reduction

A 48-year-old man presented to the emergency department after falling onto his left hand while intoxicated. He had a history of psoriasis treated with topical medications only, with no other medical conditions of note. On physical examination, the left wrist showed swelling and deformity, with no overlying skin disruption, indicating a closed fracture. Radial artery pulsation was intact. Wrist range of motion was difficult to assess due to pain; however, finger motion was preserved, and no sensory deficit was observed. Plain radiographs demonstrated a Colles-type distal radius fracture with a prominent radial volar third fragment, and closed reduction was attempted on the day of injury. Preoperative radiographs are shown in Figure 1.

**Figure 2:**
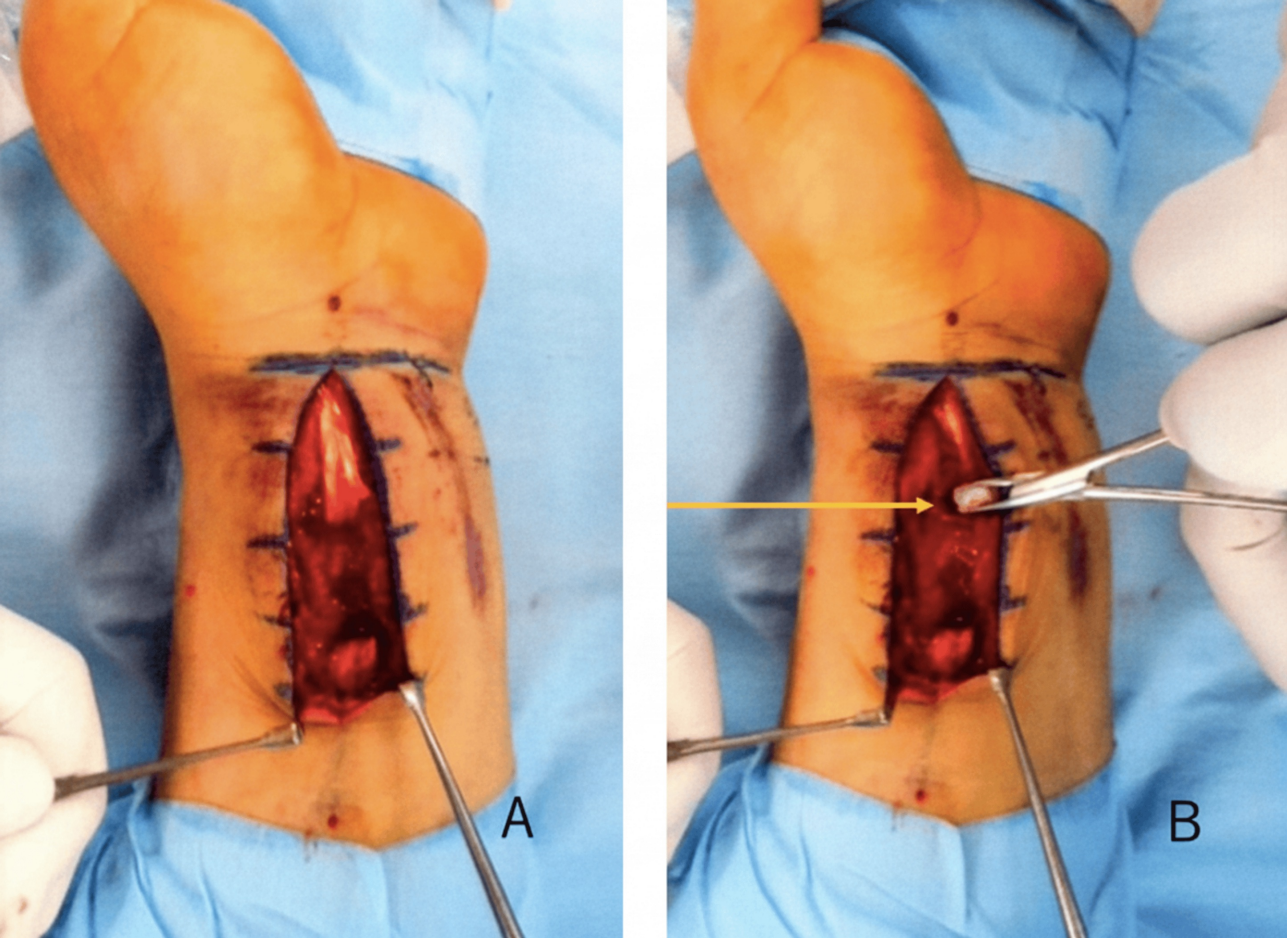
Intraoperative findings of FCR tendon rupture A: Intraoperative photograph obtained through a volar approach demonstrating complete rupture of the FCR tendon. B: Disrupted forearm fascia and pronator quadratus at the distal stump (arrow) FCR: flexor carpi radialis

**Figure 3:**
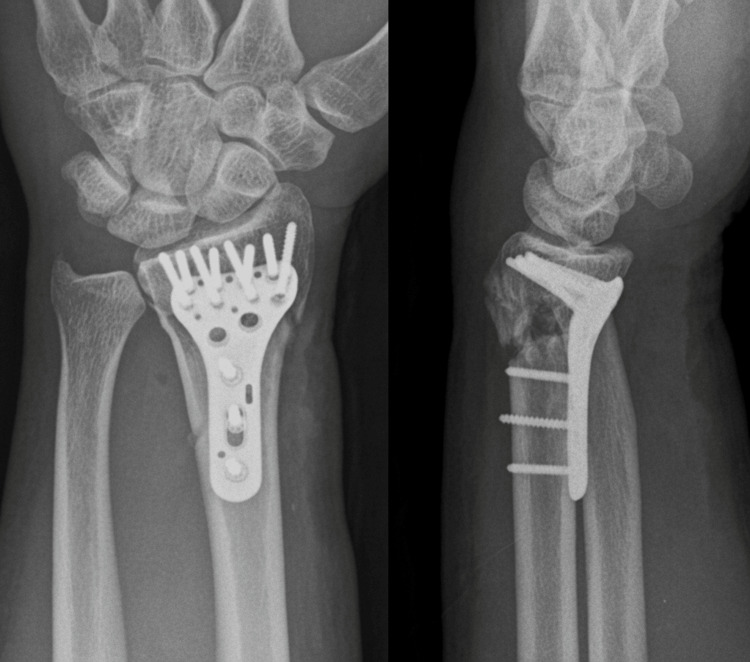
Postoperative radiographs after volar plate fixation Postoperative plain radiographs demonstrating fixation with a volar locking plate and restoration of the appropriate alignment of the distal radius

Operative treatment was performed on the fourth day after injury. Through a volar approach, complete rupture of the FCR tendon was identified during subcutaneous dissection. At the distal stump, the forearm fascia and pronator quadratus were disrupted (Figure 2), and a sharp volar bone fragment was protruding beneath the tendon. After fixation using a volar locking plate, postoperative radiographs confirmed appropriate alignment (Figure 3). The FCR tendon was repaired using a four-strand modified Kessler technique with additional running sutures. 

Postoperatively, splint immobilization was maintained for two weeks, followed by the use of a wrist support. Finger motion was allowed immediately after surgery. Wrist range-of-motion exercises, excluding extension, were initiated at two weeks, and wrist extension was permitted at four weeks. The patient returned to work without restriction at three months. At five months postoperatively, grip strength on the affected (non-dominant) side was 30 kg, compared with 34 kg on the contralateral side. Wrist range of motion was nearly symmetrical, with extension of 75° (contralateral 85°), flexion of 85° (90°), and full pronation and supination of 90° bilaterally. The DASH (Disabilities of the Arm, Shoulder, and Hand) score was 3, indicating minimal residual disability. Overall, the patient achieved near-complete functional recovery.

**Figure 4:**
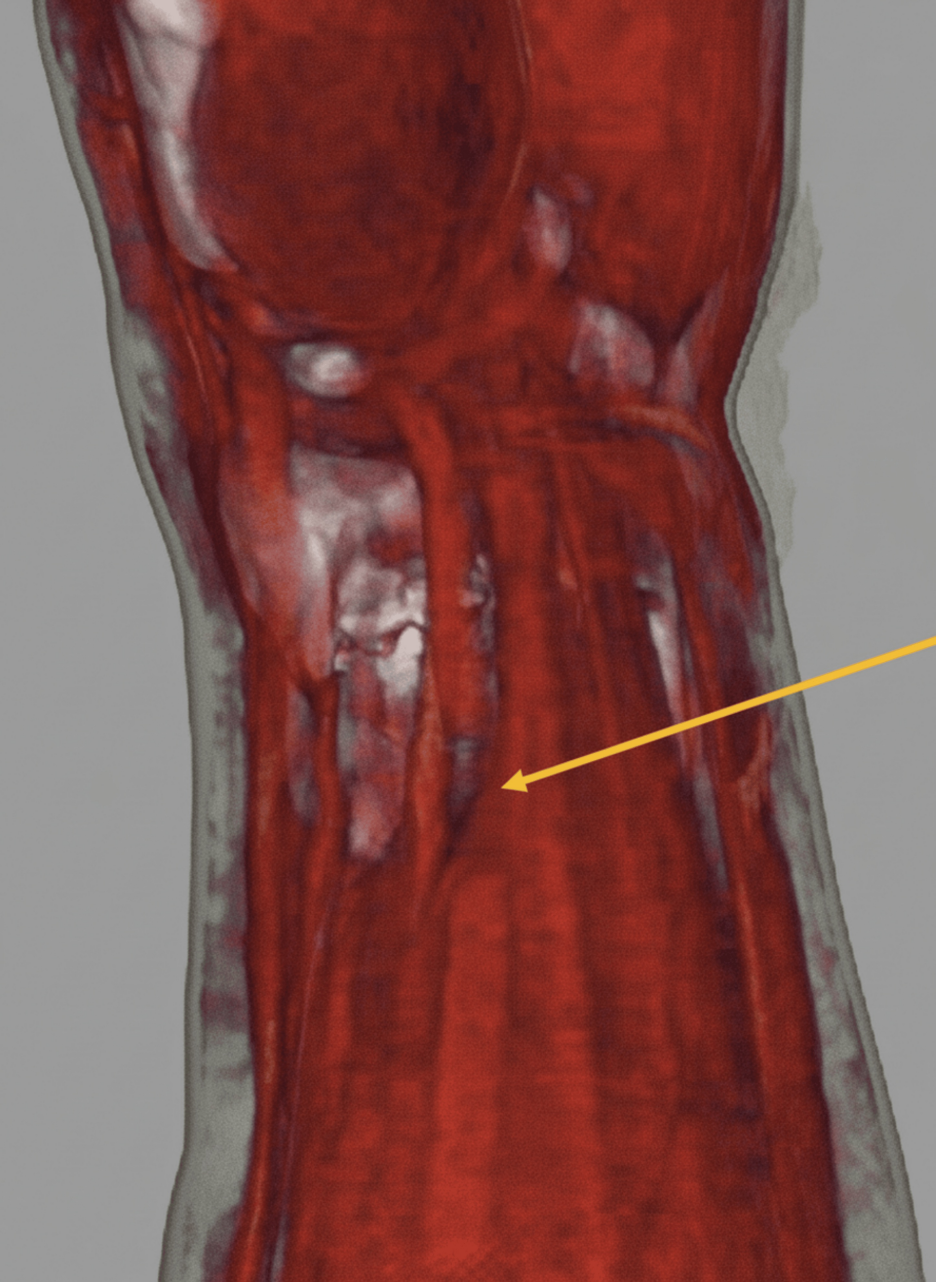
Three-dimensional CT reconstruction showing FCR tendon discontinuity Retrospective three-dimensional (3D) reconstruction of preoperative CT images using soft-tissue settings demonstrating discontinuity of the FCR tendon (arrow) corresponding to the level of the volar bone fragment CT: computed tomography; FCR: flexor carpi radialis

To assess whether the FCR tendon rupture could have been detected preoperatively, retrospective three-dimensional (3D) reconstruction of preoperative CT images using soft-tissue settings was performed, which clearly demonstrated discontinuity of the flexor carpi radialis tendon corresponding to the level of the volar bone fragment (Figure 4).

## Discussion

FCR tendon rupture at the time of distal radius fracture is extremely rare, with only five cases reported in the literature [4-8]. In all reported cases, including the present case, the rupture was discovered intraoperatively during fracture fixation [4-8]. Additionally, previously reported cases have tended to occur in relatively younger patients sustaining moderate-energy trauma, similar to the present case [4-8]. In cases where primary repair was performed, postoperative outcomes were favorable, with no significant functional deficit [4,5,7,8], whereas in one reported case without tendon repair, residual limitation of wrist motion was observed [6].

Despite these outcomes, early diagnosis remains a significant challenge. Typical clinical findings of FCR rupture include localized swelling, tenderness, a palpable defect, and weakness in wrist flexion and radial deviation [3]. As wrist flexion and radial deviation are functions shared by multiple muscles, these movements may be preserved even with FCR tendon rupture, making clinical diagnosis difficult. Furthermore, in cases associated with distal radius fracture, evaluation is often difficult due to severe pain, swelling, and limited active motion, making clinical evaluation nearly impossible. Consequently, preoperative diagnosis has not been achieved in previously reported cases [4-8].

The key clinical message from this case is the strong association between the injury mechanism and the specific fracture pattern. A common radiographic feature across reported cases is the presence of a volar protruding bone fragment [4-8]. In our case, a prominent radial volar third fragment was located directly along the anatomical course of the FCR tendon. This suggests that sharp volar cortical spikes or displaced fragments in this region may mechanically compromise the tendon at the time of injury. Therefore, clinicians should maintain a high index of suspicion for associated FCR tendon injury whenever a prominent volar fragment is observed.

To explore methods for preoperative detection, we retrospectively reconstructed the preoperative CT images using soft-tissue settings. The tendon course and discontinuity were clearly visualized. The reconstruction process was straightforward, required minimal operator input, and was reproducible. MRI remains the gold standard for assessing soft-tissue injuries, such as tendon or ligament tears, or occult fractures [9-12]. However, MRI is less frequently considered or performed in the acute assessment of distal radius fractures. In contrast, CT is often obtained for detailed fracture characterization and preoperative planning. In this context, our case suggests that appropriately reconstructed 3D CT images can clearly visualize tendon discontinuity without additional imaging burden or significant operator input. While MRI should be considered if independent soft-tissue injuries are suspected, 3D CT may offer a highly practical and reproducible diagnostic advantage for detecting tendon ruptures directly caused by fracture fragments.

## Conclusions

We described a rare case of FCR tendon rupture associated with a distal radius fracture. In relatively young patients with distal radius fractures demonstrating a volar protruding bone fragment, associated FCR tendon rupture should be strongly suspected. 3D-CT reconstruction using soft-tissue settings provides a valuable and accessible tool that may facilitate preoperative recognition of tendon discontinuity. Awareness of this injury pattern and the potential utility of 3D-CT may improve surgical preparedness and patient counseling.
